# Assessing the Effects of COVID-19 on Restaurant Business From Restaurant Owners’ Perspective

**DOI:** 10.3389/fpsyg.2022.849249

**Published:** 2022-04-15

**Authors:** Sazu Sardar, Rudrendu Ray, Md. Kamrul Hasan, Shital Sohani Chitra, A. T. M. Shahed Parvez, Md. Ashikur Rahman Avi

**Affiliations:** ^1^Department of Tourism and Hospitality Management, University of Rajshahi, Rajshahi, Bangladesh; ^2^Department of Marketing, University of Rajshahi, Rajshahi, Bangladesh; ^3^Faculty of Business Studies, BGMEA University of Fashion and Technology, Dhaka, Bangladesh; ^4^Institute of Business Administration, University of Rajshahi, Rajshahi, Bangladesh; ^5^Department of Tourism and Hospitality Management, Pabna University of Science and Technology, Pabna, Bangladesh

**Keywords:** COVID-19, restaurant business, pandemic, mitigate, thematic analysis

## Abstract

**Purpose:**

The main purpose of this study is to assess the effects of COVID-19 on the restaurant businesses of Bangladesh. It examines the socio-economic impacts of the humanitarian disaster of the COVID-19 pandemic from the perspective of restaurant owners. The study also intends to provide recommendations to mitigate effects on the restaurant business.

**Design/Methodology/Approach:**

A qualitative research approach was adopted to explore the effects of the COVID-19 pandemic on the restaurant businesses of Bangladesh. A total of 22 in-depth interviews were conducted with the top-ranked restaurant owners in the Rajshahi City Corporation area of Bangladesh. Thematic analysis technique was applied for analyzing the collected data.

**Findings:**

The study found that the restaurant owners were compelled to reduce their number of employees, and forced to ensure social distancing and proper sanitization which increased the expenses. Although online orders and home delivery services have increased significantly, restaurant business operation is gradually turning critical owing to the lack of financial support. Thus, restaurant owners urge the govenrment for financial assistance.

**Research Limitations/Implications:**

Since this research only focused on one city in Bangladesh, the outcomes of study may have a dearth of generalizability. Hence, the investigators are encouraged to extend the study area.

**Practical Implications:**

This study will help restaurant owners and policymakers in formulating contemporary business policies and strategies. Thus, it will be supportive of improving the present condition of the restaurant businesses in developing countries like Bangladesh.

**Originality/Value:**

This paper identified the truculent scenario of the restaurant business during the COVID-19 pandemic.

## Introduction

The COVID-19 (coronavirus) pandemic affects the human race and stimulates a universal calamity. The pandemic has very perceptible effects on the tourism and hospitality industry, as this industry was hit the hardest (World Travel and Tourism Council (WTTC), 2020) when communication and transportation systems, especially airlines, ceased their service globally. There is no other sector overturned as severely as tourism and hospitality industry because of COVID-19 (Zhang et al., 2021) and the effects were direct and devastating. The Statistical, Economic, and Social Research and Training Center for Islamic Countries (SESRIC, 2020) reported that out of 217 destinations in the world, 65% are closed for tourists while 25% imposed travel restrictions on the travelers of some specific countries (SESRIC, 2020). Qiu et al. (2021) mentioned that the Asia Pacific region was first affected by COVID-19, followed by the North American region, Europe hereafter, and finally the Africa region. As a result, almost all countries closed their borders up to March 2020 and it almost collapsed the international tourist arrivals which had a high (95–100%) impact on their economy and it was forecasted that the pandemic will cause a loss of around 50% in the year 2021 (Fotiadis et al., 2021). Thailand lost 6% of its GDP in 2020 as the country is mostly dependent on tourism income (Qiu et al., 2021). The pandemic has affected tourist flow negatively causing the industry to lag behind by 15 years due to COVID-19 (Fotiadis et al., 2021) and it will take at least 1 year to recover the losses partially and 4 years to recover fully (Zhang et al., 2021).

Bangladesh has also experienced an adverse impact on tourism and hospitality-associated businesses. When the virus came to Bangladesh, the government declared a state of emergency and there was chaos in the country. Like other countries around the world, the way of life has changed drastically where Bangladesh also lost the traction of business. There was an adverse impact on tourism and hospitality-associated businesses. According to the report of the Bangladesh Tourism Board (BTB, 2021), the sector lost 1487.02 Crore BDT because of the pandemic. Since the industry is made up of several sectors, the crisis began with the restaurant sector because people began to avoid large gatherings, especially restaurants, as the virus was initially also transmitted *via* food, needless to say, this severely affected the restaurant business. This study is an attempt to explore the effects from the perspective of restaurant owners.

### Literature Review

Jaipuria et al. (2021) conducted a study to forecast the inbound tourist arrival rates and foreign exchange earnings (FEE) in India. The study is introduced with a few statements of mention, such as how the COVID-19 pandemic reduces the FEE, hinders regional developments, destroys job opportunities, and breaks down host communities’ confidence. Thus, the study tried to predict the consequences of the pandemic on the tourism industry of India. The study used Artificial Neural Networks (ANN) as an effective method for prediction purposes. It is shown that tourist arrivals decreased by 6.63% in February and 66.42% in March because of border closure, international flight cancellation, and series of lockdowns. It is also confirmed that from August 2020 to September 2021, the Indian tourism industry could earn at least 31,325.25 million USD despite COVID-19 situation. The study forecasted that the monthly FEE would be 1,790.53 million USD. However, the rate of monthly arrival may fall to 68%. The study also found that proper management of tourism activities could generate the FEE USD 13,351.07 million while in a vice versa situation, the FEE may fall below USD 1,790.53 million with entire losses. The authors argued that tourism generates employment opportunities as well as increases revenue. But there is a chance of losing 9 million jobs in India due to the pandemic. So, it is recommended to adopt proper strategies to restructure the industry, earning USD 1,790.53 million FEE, utilizing resources and creating employment opportunities. The authors are hopeful that this study will contribute as a foundation study and will help decision-makers to take the prompt decision, promote destinations, subsidize the industry, implement effective health protocols, and design an up-to-date tourism and hospitality curriculum. The authors also stated a few limitations of this study as it is conducted based on the inbound tourist data of only one country- India, which may extend to other countries and may consider domestic and outbound tourists.

Another impact-based study was conducted by Foo et al. (2020) in Malaysia. The study highlighted the impact of the COVID-19 pandemic on the airline and hotel business besides discussing the supportive packages of the Malaysian government. In the case of impact on airline industry, the study reported that the top three airlines- Malaysian Airlines, Malindo Air, and Air Asia cut the salary of employees ranging from 10 to 100% depending on position, where some employees also left their jobs without any payment. The airlines lost revenues and may fall into bankruptcy, therefore urging the government for financial support. On the other hand, COVID-19 caused loss of RM 68,190,364 in revenue as 170,084 hotel room bookings had been canceled since 11 January 2020 to 16 March 2020. Among the cities, Kuala Lumpur lost the most revenue (RM 23,021,301) and Sabah lost second highest revenue (RM 11,550,605). It is forecasted in the study that the local hotel sector will lose RM 3.3 billion by June 2020. These huge losses forced the hotel owners to take a drastic yet inevitable decision with the workers’ jobs and salaries. The study surveyed 17,826 workers and it reported that 3% employees were laid off their jobs, 20% faced unpaid leave and 16% faced salary cut. The Malaysian government took immediate action declaring several stimulus packages for the affected stakeholders to help in boosting the tourism industry. The packages include flexible guidelines for hotel use, 15% discount on electricity bills, exemption of taxes and other utility payments, raising Human Resource Development Fund (HRDF), restructuring bank loans for 6 months, and wage subsidy programs. The government also took timely initiatives to promote domestic tourism. Personal income tax relief, digital vouchers for transportations (Air, Rail) and hotel accommodation are implemented in this regard. Thus, the authors believed that such initiatives will help the country to recover the losses and boost the tourism industry.

With the spread of COVID-19, hotel guests are now more concerned about health and hygiene. Thus, authors (Fotiadis et al., 2021) recommended reshaping or reassessing the present business model and adopting innovative steps for restructuring the confidence of the hotel guests/customers. Sharma et al. (2021) completed a study to evaluate the effectiveness of such innovations from the shareholders’ perception. The study assessed four categories of innovation-product, process, organizational and marketing developed by the Oslo Manual and also examined the innovation success through the life cycle model of innovation process developed by Schumpeter (1934). The study highlighted that from 20 February 2020 to 18 June 2020, six chain hotels (Hyatt, Marriot International, Wyndham, Hilton, Choice, and InterContinental) announced four types of innovations. Among the hotels, Marriot International adopted innovations strategies in seven times and the hotel adopted organizational innovation four times, and product-process innovation three times. On the other hand, InterContinental Hotels only adopted organizational innovation once. Among the innovation categories, the organizational and product-process innovation received more attention whereas marketing innovation has less attention. The study also analyzed the impact of such innovations on hotel market value and found positive influence. It is mentioned that innovative strategies reduced the risk of panic of the guests and hotel employees. The test of this study proved that product-process innovations have a more vital influence on shareholders’ perception than organizational and marketing innovations. The study advised hotel industries to carefully implement the innovations as the innovations pose a positive influence on the hotel’s strategic and operational performance. They also mentioned some limitations. Firstly, they considered only privately traded hotels. Secondly, the study partially applied *ex ante* analysis and finally, the study used the fundamental neoclassical economics principles of market efficiency-based framework (analytical Framework).

Fu (2020) conducted a study to examine the impact of COVID-19 and outline the strategies to recover the losses from Taiwan’s hospitality industry perspective. The study analyzed secondary data which was collected between the pandemic and non-pandemic periods. The analysis revealed that during the pandemic periods, there was a decrease in occupied rooms number, occupancy rates, operational revenue and employees’ numbers in the hotel sector of Taiwan. Specifically, the hotels in the northern region of Taiwan suffered higher loss in their revenue. Room occupancy rates were highly decreased in this region. The study also found that the Transportation and Communication Ministry extensively supported this sector. The government approved a total of NT $7 billion in supporting tourism-related sector as the means of relief, subsidies and revitalization. The subsidized amount is also utilized to maintain the hotel’s operational expenses and paying employees’ wages. The banks also sanctioned soft loan facilities for the tourism and hospitality industry. It is noted that the authority also increased their marketing activities to attract foreign tourists, and introduced reward schemes for the tourist, local government, and tourism associations. The M.O.T.C also subsidized NGOs for promoting and inventing digital tourism activities and experiences. In conclusion, the authors of this study argued that the Taiwan hospitality industry faced huge losses during the pandemic. The study proposed some recovery tactics for protecting the tourism and hospitality industry. The proposal includes enhancing risk and crisis management awareness programs such as training, establishing a crisis management agency, Big Data analyses and applying Artificial Intelligence.

Higgins-Desbiolles (2020) marked COVID-19 as an opportunity to make tourism more responsible for the society and ecology. Thus, this conceptual paper offered several thoughts and actions to rethink and reset tourism to make it sustainable. The study first identified the COVID-19 as a serious global crisis and urged for government interventions, social safety nets and social caring. It is mentioned that the needs and interests of the host communities should be prioritized by tourists and tourism service providers. The author noted such interventions as socializing tourism, which means enhancing the empowerment and welfare of local communities through tourism. The study also appraised the government’s voluntary social, educational, and health-related interventions in response to COVID-19 and dispraised the privatization, marketization, and commercialization initiatives. The research emphasized on the development of domestic tourism and small and medium tourism enterprises for mitigating the effects of COVID-19. It is also recommended to make tourism serviced and accountable to the public. As a reset measure for the tourism industry, the study suggested substitute models in the forms of cooperatives, non-profits, social enterprises and social business so that it may facilitate tourism. Finally, the researcher stated it “must” be ensured that national tourism organizations are public service based as this will also ensure workers’ rights and a secure working environment.

The study of Qiu et al. (2020) surmised the citizens’ Willing to Pay (WTP) perception toward the risk linked with tourism activities for the COVID-19 pandemic. The estimation is done in three Chinese cities- Guangzhou, Hong Kong, and Wuhan through a quantitative survey-based approach. The study collected data from 1,627 respondents and is analyzed in comparative form. The analysis of the study reported that citizens of the three cities have a positive and indifferent attitude toward reducing the risks and they are willing to pay on average 300 of their local currency to mitigate the negative effects. The study marked it as an effect of extensive media coverage of the pandemic which made citizens aware of the pandemic. It is also found that the demand for risk reduction is more elastic in Guangzhou than the other two cities. This research analyzed the relationship between age and WTP and interestingly found that WTP tendencies are higher among the young residents than the senior citizens. In this point, the study also described that young generations gathered extensive information through social media and the internet which made them more aware about the pandemic than the other citizens. As a result, the other class of citizens has limited exposure, thus they are more conservative. In the investigation of the relationship between income and WTP, it is found that residents from Guangzhou and Hong Kong have high incomes and they are willing to pay more to reduce the risk. In the same way, tourism practitioners of Hong Kong and Wuhan want to donate more than the other residents as tourism practitioners hold superior social responsibility. The research also expressed the estimated social cost through monetary value. Accordingly, the cost tendency is most similar in the three cities. The social cost for Hong Kong is RMB 825 million, Guangzhou- RMB 1,417 and Wuhan- RMB 1,215 million. The study asserted that this research has theoretical and practical implications and tourism research will be benefited from the findings of this research. But, response biases remain a concern as the survey was conducted during the peak of COVID-19. Moreover, exploration of social cost rather than impact analyses is also identified as a limitation of this study.

Hall et al. (2020) discussed the COVID-19 pandemic and its impact on the tourism industry, country, and tourist behavior. The study also focused on the impact on transportation and networking among the countries. It is found 90% of the world population confined themselves in their homes or own country because of the closed borders and travel restrictions. The study found that the number of guests declined to 50% between 21 March 2019 and 21 March 2020. On 14 April 2020, the revenue of airlines decreased to 55% and air traffic reached only 48%. This study also categorized the political, economic and environmental impacts of COVID-19 into three parts- impact on tourism generating regions, impact on tourist destinations, transit regions and competing destinations and businesses. Interestingly, the health security protocols are becoming common to every part. Moreover, it is found that COVID-19 changed consumer and industry behavior, demotivating tourists of the tourism generating region. The COVID-19 pandemic changed the transport linkages among the transit regions while the destination marketing organizations are facing difficulties to find out the most appropriate ways to position themselves in the new normal in the mind of the tourists and highly affected destinations. The tourist destinations are obliged to the government’s regulatory and legislative actions and orders. Some of the destinations are also taking voluntary measures as they are planning and preparing themselves in mitigating the COVID-19 pandemic effects. The study mentioned that non-pharmaceutical interventions are a common response taken by the destinations. It is also highlighted that support from government and financial/non-financial institutions, promotional strategies, development of new products and market, and enhancing destination attractiveness may recover the losses and effects of COVID-19. The authors identified several factors and interventions that may buoy change and recovery in the tourism sector. The interventions and factors are public health, economic, travel restrictions, air and other networking, access to the destinations and events, policy, low carbon emission tourism policy, market size and capacity of the destination, number of tourists and non-pharmaceutical interventions and their influence on tourist behavior and practices, mental safety and security, the confidence of the consumer and perception, sustained changes, destination image, remote working, use of staff, support from government and others, reskilling the workforce, supply chain, and preparation for future crises.

Gössling et al. (2020) completed a study in comparing the effects of COVID-19 in the tourism industry with previous pandemics. They also explored the changing patterns of society, the economy and tourism due to the COVID-19 pandemic. The study mentioned that the pandemic forced countries globally to close all sectors like RMG, transportation, oil, gas, and even educational institutions which have never happened in the last 40 years. A total of 90% of the world population were forced to confine to their homes as governments declared movement control orders to subdue the pandemic. It is mentioned that tourist arrivals declined to 20–30% in 2020 compared to 2019. But it was forecasted that the tourist arrival will grow to 3–4% in 2020. The loss is much larger and higher compared to other sectors. The study also highlighted the case of New Zealand airlines which reported that the airlines operated the flights using less than 50% of its capacity. The tourists or guest number has fallen by 50% or more all over the world. The research also found that 90% of business meetings, hotels, campsites, gastronomy, car rental activities and destinations were almost closed by March 26, 2020. On the same date, 65% of tourism businesses faced difficulties to pay their invoices whereas 72% of cafe restaurants, 63% of hotels, and 55% of Destination Management Organizations (DMOs) faced liquidity problems. The study found that 12.6 million employees of the accommodation and food service sectors are in a vulnerable position to lose their job. Major events like the Olympic Games and Euro Cup-2020 were canceled which deprived countries from profit. Thus, the research highlighted a report by UNWTO which projected that the world lost US$300-450 billion tourism receipts. The authors mentioned that restaurants were facing difficulties to recover the losses while cruise ship operators are anticipating vaccine shots where risk-free tours could be offered to vaccinated tourists. The research recommended to place emphasis on the development of the domestic tourism market. They also urged tourism sectors to see COVID-19 as a chance to reshape the industry to develop sustainability and resilience besides becoming a zero carbon emission industry.

Karabulut et al. (2020) focused on exploring different ways of measurement of the effects of pandemics through a newly developed index. The authors claimed that they are the first innovators to use the new index, and Pandemic Index Data is available in 4 months’ intervals. When analyzing the data, the authors found a negative relationship between the pandemic and tourist arrival. The spread of pandemic demotivates the tourist to travel. Moreover, different travel restrictions also act as the reduction of tourist arrival. This paper collected data from 129 countries and it is highlighted that the negative effects exist in lower-income countries. Thus, the lower-income group countries are highly deprived from tourist arrival compared to the advanced countries because of the fragile health infrastructure and corruption. The study also mentioned that the per capita GDP and exchange rate have a positive effect on tourist arrival. Tourist arrival is rising with the increase of per capita GDP and profitable exchange rate. Lastly, the authors opined that the effect of COVID-19 is difficult to predict due to its unpredictable and distinct characteristics.

### Research Gaps

The COVID-19 pandemic began to receive research attention in the middle of 2020. The uniqueness, unpredictable dimensions and devastating characteristics of the pandemic force researchers of diversified fields to examine the impacts it brings. There is a high probability that there have not been any other issues except the COVID-19 pandemic which have received such prompt attention not only from researchers but also from all levels of society in the last 5 years. It is discovered from the literatures that forecasting-based studies on COVID-19 are now common (Qiu et al., 2021; Zhang et al., 2021) and impact-based studies are also not rare but they focus broadly on the tourism and hospitality industry (Fu, 2020; Hall et al., 2020; Higgins-Desbiolles, 2020; Karabulut et al., 2020; Fotiadis et al., 2021). Again, the majority of the previous studies (Foo et al., 2020; Zhang et al., 2021) were conducted in the context of advanced countries. Meanwhile, the effects of COVID-19 are more extensive in developing or emerging nations (Karabulut et al., 2020) like Bangladesh. So, research should also focus on Least Developed Countries (LDCs).

Moreover, the second wave of COVID-19 arrived with a more destructive outlook. The wave is said to be so impactful that it is “to slay the slain.” As the wave emerged at the end of March 2021, its effects on the tourism and hospitality industry are still unexplored. The industry is considered as an umbrella concept (Walker, 2012) and associated sectors such as lodging, restaurants, airlines, tour operators, travel agents etc., are directly involved with the industry. COVID-19 has a significant influence on each of these associated sectors. Therefore, it is crucial to investigate the specific phenomenon. In this COVID-19 pandemic, restaurant businesses are faced with the most vulnerable situation, as some are forced to shut down their business and switch to other jobs where these research are unexplored. Still, now, there are no guidelines about how they rejuvenate their business. Therefore, this study will fill the gap in exploring the effects and provide suggestions to adapt in the new normal.

### Objectives of the Study

The overall objective of this study is to assess the impact of COVID-19 on the restaurant business in Bangladesh. Specific objectives are -

1. To identify the factors that affected the restaurant business of Bangladesh during the COVID-19 pandemic.
2. To assess the socioeconomic impacts of COVID-19 on the restaurant business.
3. To provide recommendations to mitigate the COVID-19 impacts on the restaurant business.

## Research Method

### Nature of the Study

This study is qualitative in nature. A qualitative model was applied to collect data through the semi-structured in-depth interview from the restaurant owners. The proposed study is a new research area in Bangladesh. Thus, the qualitative model is suitable as the new research areas are supported by the qualitative model (Mallat, 2007). Another rationality of applying the qualitative model is to explore the factors and variables that were not recognized before (Hossain, 2013). The model has the ability to capture and understand real insights, factors and variables from the contexts of the studied phenomenon.

### Sample Selection

This study identified the respondents through purposive sampling. The reason for adopting the qualitative research technique is to select respondents or sites in purposeful manner that will best help the researcher to understand the research problem (Creswell and Creswell, 2017). This study selected a sample that has available knowledge, experience about the subject, and those who were easily reachable (Berg, 2004) during the pandemic. Generally, the qualitative research sample size should not be too large that would cause the researchers to face difficulties to extract the data (Onwuegbuzie and Collins, 2007). Again, the sample should not be too small as it will be difficult to retrieve the exact data (Sandelowski, 1995), theoretical saturation or informational redundancy (Rapley, 2014). Lincoln and Guba (1986) suggested that data should be collected until redundant information occurs. This is a cyclical process of data collection and analysis that continues till the new data are found (Punch, 2013). By following the above technique, this study interviewed a total of 22 sample respondents (restaurant owners) who were interested (please see Appendices 1, 2) in participating in the in-depth interview at the Rajshahi City Corporation area of Bangladesh.

### Data Analysis Procedure

This research adopted the thematic analysis technique to analyze the collected data. Braun and Clarke (2006), and Fereday and Muir-Cochrane (2006) developed the technique. The technique helps to accumulate or merge similar opinions or comments of the respondents in the Microsoft Excel and Word document (Ray, 2019). As a result, themes and sub-themes are developed to sort and restructure the unstructured data (Ose, 2016). It is noted that the study excluded interviews 2, 8, 10, 12, and 16 as the following 17 interviews possessed all the themes and subthemes related to this study (Table 1).

**TABLE 1:** Factors and variables regarding the effects of COVID-19 on the restaurant business.

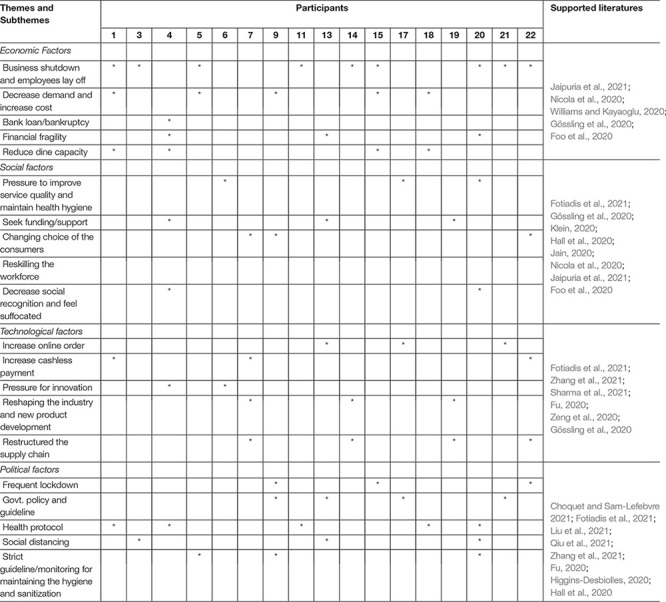

(*) Indicates response of the marked participant(s).

### Findings

**FIGURE 1:**
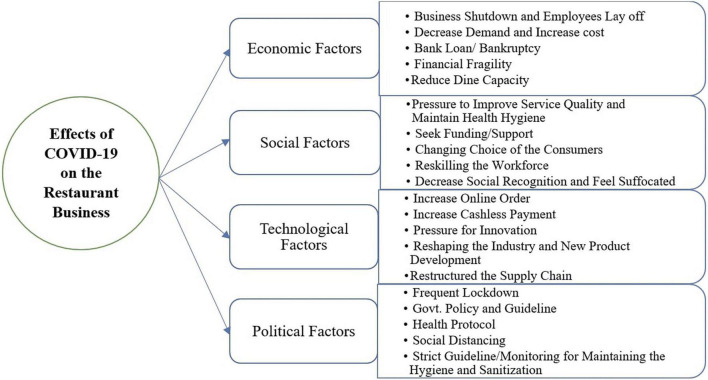
Effects of COVID-19 on the restaurant business.

The main objective of this study is to assess the effects of COVID-19 on the restaurant businesses in Bangladesh. A qualitative research approach has been adopted to explore the effects of the ongoing pandemic on restaurant businesses. Thus, the study found several variables and categorized them into four factors through the thematic analysis technique: (i) Economic Factors, (ii) Social Factors, (iii) Technological Factors, and (iv) Political Factors that are exaggerated on the restaurant business during the COVID-19 pandemic. These factors forced the restaurant owners to generate changes in their business (Figure 1).

## Discussion

### Economic Factors

This study listed the opinions (sub-themes) of the respondents as the economic factors (theme) which affect the economic condition of the business, owner and employees. Respondents highlighted that the COVID-19 pandemic crippled their business. When the pandemic first spread, employees lost their job (Table 2), restaurant owners were forced to shut down their business, and were hopeful to reopen the restaurants after a short break. But day by day the situation was more severe. When the government reopened other businesses, they did not grant permission to open the restaurants. As a result, they lost their economic strength, and did not pay salary to their employees nor pay off loan installments. For instance, one restaurant owner said-

**TABLE 2:** Employment status before and after the pandemic in the restaurants of the study area.

Total number of restaurants	240
Number of employees employed before the pandemic	1,600
Number of employees employed after the pandemic	814
Total number of employees lost the job	896

*Source: Bangladesh Restaurant Owners Association, Rajshahi District Branch.*

*I lost all of my saved money and business capital as I had not any other income source during the pandemic. I took out a loan from a local NGO just 2 months ago when the pandemic occurred. But I failed to pay the installments (Interview No. 04)* (Table 2).

The respondents also mentioned that they are facing a new reality after getting permission to open the restaurant. The demand has dramatically decreased but the operating cost has increased as several new cost heads occurred. It also decreased the profit of the business. The owners stated that-

*Students are our main consumers. But they are now staying home as the educational institutions including the dormitories are closed. So, we lost the new normal demand. Moreover, we have to allocate money for the newly incurred costs such as purchasing masks and hand sanitizer for the employees and the consumers (Interview no. 01, 05, 15 and 18).*

The findings of this section are reflected in the study of several scholars. Nicola et al. (2020) and Williams and Kayaoglu (2020) found that COVID-19 makes life more challenging for temporary workers while 50 million jobs in the tourism sector are at high risk. The situation is more devastating in the least developed countries and restaurants are closed in most of the developing countries (Foo et al., 2020; Gössling et al., 2020; Jaipuria et al., 2021).

### Social Factors

The pandemic also changed the attitude and behavior of the people/consumers of our society (Gössling et al., 2020). The variables of such changes are accumulated as the social factors. Respondents opined that the present consumers are more concerned to maintain hygiene and sanitation. They prefer to sit while maintaining social distance. As the consumers are more particular in COVID-19 prevention measures, the restaurants are also highly concerned about quality and hygiene. The restaurant mentioned-

*When our customers enter our restaurant premises, they ask for hand sanitizer or they use their personal sanitizer. The customers also guide our staff to wear masks and set tables three feet away (Interview No. 06).*

*I observed that customers are preferred to take one-time glass and beverage cans. Even they do not want to share the glass and can with their partners (Interview No. 22).*

The respondents mentioned that they need to reskill their employees with the changed behavior of the consumers. They feel in a suffocating position as they are frustrated by the pandemic. Their social dignity is also fragile along with their economic strength. As an example-

*I don’t know when the situation will be normal but it should happen soon. I lost my hopes and social dignity. Now, my family expenses are borne by my father-in-law. I feel dishonor but I have nothing to do (Interview No. 20).*

*As I am failing to pay the installments, the officers of the NGO regularly visit my residence for the payment and I leave before their arrival (Interview No. 04).*

The above findings are supported by some literature (Foo et al., 2020; Gössling et al., 2020; Hall et al., 2020; Jain, 2020; Klein, 2020; Nicola et al., 2020; Fotiadis et al., 2021; Jaipuria et al., 2021).

### Technological Factors

Despite several negative effects, technology has progressed during the COVID-19 pandemic. The pandemic motivates managers to use artificial intelligence and robotics in their business operations (Zeng et al., 2020). Respondents of this study mentioned that online order and app-based food delivery services gained more popularity in the pandemic. Moreover, the government encouraged home delivery instead of dining-in in the restaurants. It puts pressure on restaurant owners to develop innovative strategies and build on product development. The participants said-

*Before the pandemic, I served food in my restaurant. But during the pandemic, I made contact with the Food Panda and Food Shahi apps-based food delivery services. Now, I am running my business through the apps (Interview no. 13, 17, and 21).*

*As customers are more active in social media during this pandemic, I am running promotional activities through Facebook (Interview No. 14).*

*At present, it is needed to build a network with the online service providers and Banks. They appeared in my business as a new supportive entity. Every week I visited 3 banks to collect the payment paid by customers through mobile banking, credit card, or debit card (Interview nos. 07 and 19).*

The studies by Fu (2020), Gössling et al. (2020), Zeng et al. (2020), Fotiadis et al. (2021), Sharma et al. (2021) and Zhang et al. (2021) supported the discussed findings of the technological factors.

### Political Factors

In the COVID-19 pandemic, government intervention is the common scenario for all sectors. Governments imposed different rules, regulations, and guidelines to control the spread of the virus. But sometimes, such interventions are not implemented properly due to the lack of proper monitoring and contemporary decision which negates its efficacy. Respondents highlighted that the government did not implement the lockdown properly, which caused the virus to spread out more extensively. As such, the aftermath has caused a longer period of lockdown where restaurants were forced to shut down, thus destroying the business. The owners mentioned that-

*The government only thinks about the industrialists. As we are small, we are out of consideration (Interview No. 09).*

*Local administration only forced us to strictly follow the health protocols, social distancing and hygiene. But we rarely get any financial assistance from them during the pandemic (Interview No. 20).*

Various research (Fu, 2020; Hall et al., 2020; Higgins-Desbiolles, 2020; Choquet and Sam-Lefebvre, 2021; Fotiadis et al., 2021; Liu et al., 2021; Qiu et al., 2021; Zhang et al., 2021) also mentioned different interventions of the government.

### Implications of the Study

#### Theoretical Implications

This research incorporates a new field of study (COVID-19) and contributes to the existing literature and body of knowledge. The uniqueness of this study is that the variables are extracted from the participants whose business have been affected directly because of the pandemic. Furthermore, the variables are also supported by extant literatures. The researchers could consider the factors and variables as a basis of further casual research or quantitative study. The identified variables could also be used for developing a conceptual model. Again, it is found from the literatures that impact based studies on COVID-19 and restaurant business are rare. Thus, this study is viewed as a unique contribution in the field of tourism.

#### Practical Implications

The COVID-19 pandemic affected not only human beings but also has diverse effects on each sub-sector of the tourism and hospitality industries. As mentioned in the research gap section, extant literatures make evident that an investigation should be made on this specific phenomenon. The major contribution of this study is that it is fully focused on the restaurant business which is one of the major sub-sectors of tourism and hospitality industry. The research identified the factors which affected restaurant entrepreneurs during COVID-19. One of the notable parts of this research is that the findings are the inner reflection of the affected people as the data is collected from restaurant owners, who are the directly influenced group. But it is important first to know the major effects and influencing factors for mitigating the effects. This study identified the factors and effects of COVID-19 on the restaurant business. Thus, the identified factors could guide the entrepreneurs to handle the present and future pandemic situation. Therefore, the findings of this study have significant implications for Restaurant Owners Association of Bangladesh, Ministry of Industries, Ministry of Civil Aviation and Tourism, Non-government Organizations (NGOs), Small and Medium Enterprises (SMEs), and other policymakers in Bangladesh as well as other developing countries.

### Conclusion

This study explored the impacts of the COVID-19 pandemic on the restaurant business. The findings of this research presents the practical and lived experience of the restaurant owners. Thus, it is found that restaurant businesses are affected in four aspects which are the economic, social, political, and technological aspects during the COVID-19 pandemic, the economic aspect being the most severe. It is also found that dramatic changes like rise of online delivery and social distancing ocurred in the business, greatly challenging restaurant owners. Nevertheless, they have to run their businesses and are managing and adapting to all the changes. They urgently need cooperation and assistance to mitigate the effects. Thus, this study proposed five measures/guidelines: (i) Government should support businesses with the cooperation of financial assistance. (ii) The central bank could order all the banks to render soft loan/interest-free loans to the restaurant business. (iii) In addition, the restaurants should be exempted from all types of taxes for the next 3 years. (iv) As the pandemic caused changes in various aspects, the owners, managers and employees should be trained to cope with the change. Moreover, the restaurant owners should also maintain the health protocol and hygiene to prevent the spread of the virus.

This study will help restaurant owners and policymakers in formulating contemporary business policies and strategies. Thus, it will be supportive to improve the present condition of the restaurant businesses in developing countries like Bangladesh. The study clinches with few limitations. Firstly, the area of the study is small which may not represent the total restaurant business’ opinion. Secondly, there may be some other factors and variables which may also influence the restaurant business. Thirdly, the study only considered the opinions of the restaurant owners. The opinions may differ in the case of other stakeholders such as managers, employees, and customers. Finally, the study followed the qualitative model. The model does not quantify or measure the opinion of the respondents. Thus, the quantitative model may be applied to test the significance of the opinion. The authors are hopeful to overcome the limitations in future studies.

## Data Availability Statement

The original contributions presented in the study are included in the article/supplementary material, further inquiries can be directed to the corresponding author/s.

## Author Contributions

SS initiated, coordinated, and completed the research manuscript and had a remarkable contribution to the literature review. RR, MH, and AP contributed to the data collection and research methodology section. SC, AP, and AV contributed to semi structured questionnaire design and data analysis. All authors contributed to the article and approved the submitted version.

## Conflict of Interest

The authors declare that the research was conducted in the absence of any commercial or financial relationships that could be construed as a potential conflict of interest.

## Publisher’s Note

All claims expressed in this article are solely those of the authors and do not necessarily represent those of their affiliated organizations, or those of the publisher, the editors and the reviewers. Any product that may be evaluated in this article, or claim that may be made by its manufacturer, is not guaranteed or endorsed by the publisher.

## Appendix

**TABLE A1:** Characteristics of the restaurants.

Sl.	Name of the restaurants	Type of cuisine	Dine capacity	No. of employees (post pandemic)	Monthly profit in Tk. (post pandemic)
1	Master Chef	Multi-Cuisine	250	20	240k
2	Nanking Chinese Restaurant	Indian/Chinese	350	30	Not Mentioned
3	Tasty Time	Fast Food/Chinese	100	15	90k
4	The Hideout Cafe	Fast Food/Chinese	50	12	40k
5	Order’s Up Legacy	Fast Food/Chinese	100	10	120k
6	Chili’s Thai and Chinese	Thai/Chinese	100	20	180k
7	Khan Teheri Ghor	Bangla	60	15	70k
8	Aziz Canteen	Bangla	45	12	110k
9	Hungry Heroes	Fast Food	50	18	40k
10	Midas Restaurant	Multi-Cuisine	200	20	170k
11	S. K Food World	Multi-Cuisine	80	15	90k
12	Melting Moments	Indian and Chinese	30	10	40k
13	Amana Fun Ville	Multi-Cuisine	250	25	180k
14	Golpo Kotha Food Corner	India	50	10	40k
15	Daruchini Chinese	Indian and Chinese	180	20	100k
16	The Mahal	Indian and Chinese	60	12	50k
17	Coffee Bar	Chinese	500	50	280k
18	Ekota Hotel and Restaurant	Bangla	80	20	70k
19	Ibrahim Hotel and Restaurant	Bangla	100	20	100k
20	Rahmania Hotel	Bangla and Indian	80	15	180k
21	Nawab	Indian	12	7	40k
22	Flavours	Thai/Chinese	80	9	60k
					

**TABLE A2:** Profile of the participants.

Demographic characteristics	Frequency	Percent (%)
**Age of the participants**		
Less than 30 years	05	22.73
31–40 years	09	40.00
41–50 years	05	22.73
51–60 years	2	9.09
60 years or more	1	4.55
**Education**		
SSC level	02	9.09
HSC level	08	36.36
Graduation level	12	54.55
**Income level (per month)**		
Less than Tk. 30,000	04	18.18
30,001–35,000	06	27.27
35,001–40,000	02	9.09
40,001–45000	06	27.27
45,001–50,000	1	4.55
50,000 or more	1	4.54
**Gender**		
Male	18	81.82
Female	04	18.18
**Marital status**		
Unmarried	03	13.64
Married	19	86.36
